# Supplementary material for: The effects of plyometric training with speed and weight overloads on volleyball players’ strength, power, and jumping performance
Source: PLoS One. 2025 Feb 27;20(2):e0316477. doi: 10.1371/journal.pone.0316477 (PMC11867311; doi:10.1371/journal.pone.0316477)
Supplement: Attachment — Submitted filename: Responses to reviewers.docx [file pone.0316477.s002.docx]

We sincerely thank the reviewers for their thoughtful and constructive feedback on our manuscript. Your comments and suggestions have significantly enhanced the work's clarity, rigor, and overall quality. We appreciate the time and effort you dedicated to reviewing our submission and providing valuable insights, which allowed us to improve the manuscript and address essential aspects of the study. Your contributions are greatly appreciated.

Best wishes,

Corresponding Author

**Reviewer 1:**

| **Comments** | **Responses** |
| --- | --- |
| Please refer to the PLOS ONE standard for authors following the instructions and guidelines: abstract format, reference styles in the text, references, etc. | *They were corrected based on PLOS ONE instructions.* |
| Please add sentence/paragraph numbering at the beginning of each sentence. | *It was corrected based on the reviewer’s comment.* |
| Some references are old. Please use more recent references. | *It was corrected based on the reviewer’s comment. Of course, some of them were necessary and could not be deleted.* |
| Please add a brief description of the installation of the subjects, as technique and position are crucial in the assessment, both at isokinetic and isometric modes. | *Thank you for your valuable feedback and for emphasizing the importance of including a detailed description of the installation techniques and subject positioning during the isokinetic and isometric assessments. In response, we have added a section to the Materials and Methods under the Isometric and Isokinetic Strength Parameters subsection that comprehensively explains the subject setup during testing.* |
| The absolute peak torque is becoming increasingly rare to use in isokinetic assessments; we instead use the PT reported to body weight for greater accuracy. The Biodex provides this measure in the isokinetic parameter table. Try adding this parameter and comparing your results. | *The relative peak torque (PT normalized to body weight) has been added to the manuscript, and the results have been compared and discussed accordingly.* |
| The use of the t-test in this study seems to me to be the main problem. Indeed, a repeated measures ANOVA should be considered instead of the t-test. The interactions between groups and parameters are also interesting to analyze and observe. | *The statistical analysis has been revised, and repeated measures ANOVA has been applied instead of the t-test. Additionally, interactions between groups and parameters have been analyzed and included in the results and discussion sections.* |
| Results and Discussion are well written, but it remains to be seen whether the statistics are correct, as these two sections are totally dependent on them. | *Thank you for your observation. The statistical analysis has been thoroughly reviewed and validated to ensure accuracy, and the* Results *and* Discussion *sections have been confirmed to align with the revised statistics.* |

**Reviewer 2:**

| **Comments** | **Responses** |
| --- | --- |
| Page 10:  1. One of the other methods of using the speed variable is overspeed training (OST). This method has been used for years as a particular exercise for athletics. Which is given to the athlete at a speed higher than the maximum speed by using tilt, … (consider the grouping into one single sentence) this method is also called assisted Training. (suggestion: use quotation marks and no caps). | *Thank you for your suggestion. The text has been revised to combine the sentences into one, and quotation marks have been used appropriately for "overspeed training" and "assisted training," with unnecessary capitalization removed.* |
| Page 12:  (Including full descriptions… : no capital letter needed.  There are several observations of this type in the same paragraph. | *Thank you for pointing this out. The unnecessary capitalizations in the paragraph have been reviewed and corrected to ensure consistency and proper formatting.* |
